# Emerging Molecular Antiferroelectrics: Advances and Prospects

**DOI:** 10.1002/advs.75111

**Published:** 2026-04-02

**Authors:** Xiaoqi Li, Qianxi Wang, Xiaoyu Zhang, Yating You, Zhihua Sun, Xitao Liu, Junhua Luo

**Affiliations:** ^1^ State Key Laboratory of Functional Crystals and Devices Fujian Institute of Research on the Structure of Matter Chinese Academy of Sciences Fujian China; ^2^ Chinese Academy of Sciences University of Chinese Academy of Sciences Beijing China

**Keywords:** antiparallel dipole moments, electrocaloric effect, energy storage, hybrid perovskite, molecular antiferroelectrics

## Abstract

Antiferroelectrics (AFEs) with typical reversible electrically antipolar‐polar phase transformation have attracted tremendous attention for assembling electronic devices. As a thriving part of the family, molecular antiferroelectrics, referring to the crystalline assembly of organic molecules and organic‐inorganic hybrids, have emerged due to their diverse structures, facile synthesis, and intriguing antiferroelectricity. Especially, the polarization switch of molecular AFEs involves the weak interactions; thus, lower switching energy barriers and smaller required electric fields are expected in such soft materials, which manifest significant application potential in highly efficient and low‐consumption electronics applications. This review focuses on the current research progress in molecular antiferroelectrics, encompassing their diverse structures, antiferroelectricity‐associated properties, and application exploration, including energy storage, electrocaloric refrigeration, and electrostriction. Moreover, we discuss the current challenges and future opportunities in this exciting field. With the breaking of traditional inorganic systems, molecular AFEs provide a broad and versatile platform for boosting the multifunctionality of AFEs.

## Graphical abstract

This review focuses on the current research progress in emerging molecular antiferroelectrics, encompassing their diverse structures, antiferroelectricity‐associated properties, and application exploration, including energy storage, electrocaloric refrigeration, and electrostriction. Moreover, the current challenges and future opportunities in this exciting field are discussed.

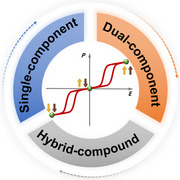

## 1 Introduction

**FIGURE 1:**
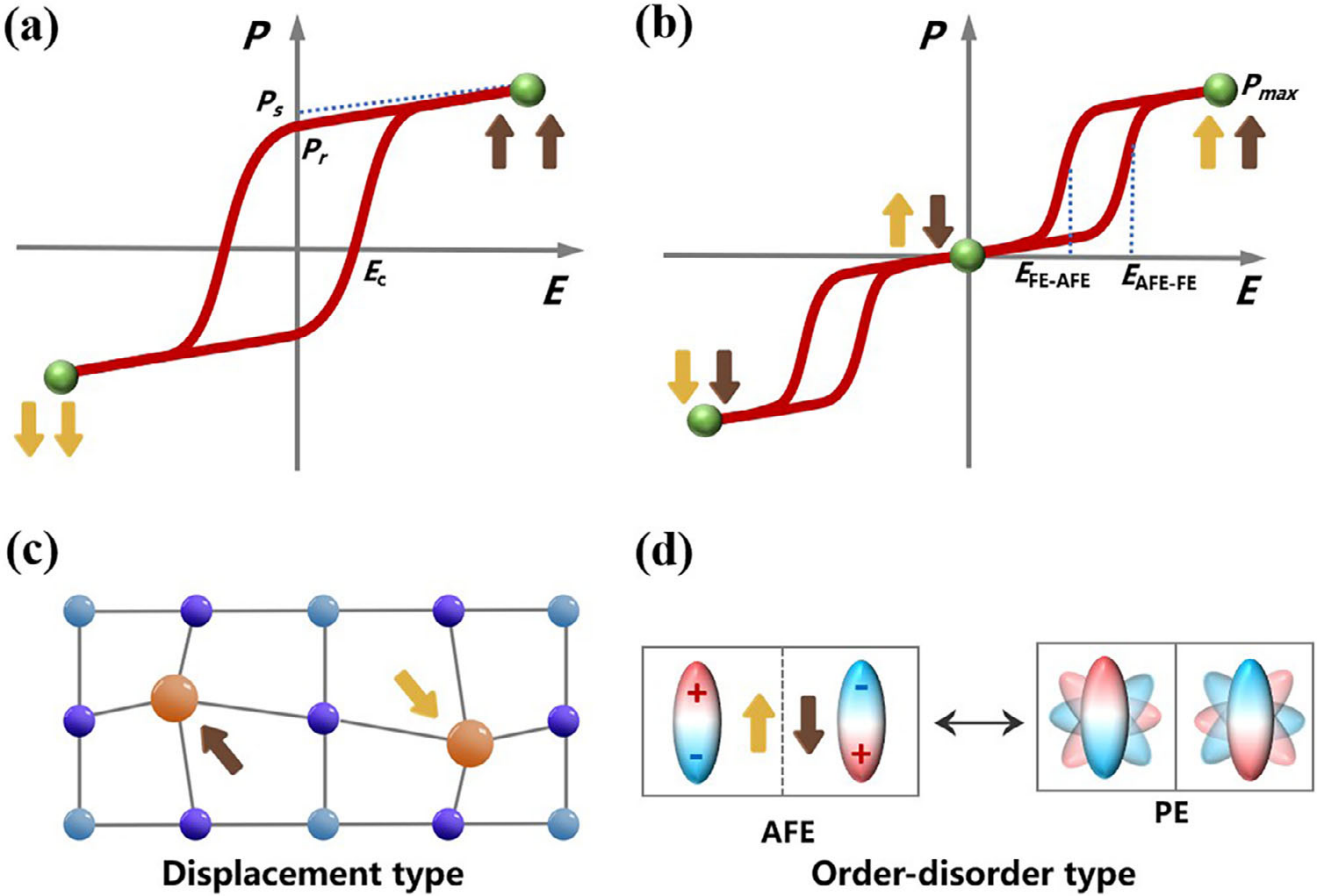
(a) Typical polarization‐electric field hysteresis loops of ferroelectrics, *P*_r_ represents remanent polarization, *P*_s_ represents spontaneous polarization, *E*_c_ represents coercive field; (b) Typical double polarization‐electric field hysteresis loops of antiferroelectrics, *E*_AFE‐FE_ represents the forward switching field from antiferroelectric‐to‐ferroelectric transition and *E*_FE‐AFE_ represents backward switching field from ferroelectric‐to‐antiferroelectric transition; Schematic diagrams of different antiferroelectric mechanisms including displacement type (c) and order–disorder type (d), the arrowheads denote the direction of dipoles.

Since the first report on ferroelectricity in Rochelle salt published in 1921, ferroelectrics (FEs) have attracted increasing attention for widespread applications in electronics and photoelectronics fields [1, 2]. FEs manifest spontaneous electric polarization, the direction of which can be switched via the application of an external electric field. Distinctively, antiferroelectric (AFE) materials do not possess macroscopic spontaneous polarization under a zero electric field, while a sufficiently high electric field can induce a large field‐driven polarization (*P*_max_) [3, 4, 5, 6]. Figure 1a,b shows the representative polarization‐electric field *P*‐*E* loops of FEs and AFEs, respectively. Antiferroelectric materials, with their unique antiparallel arrangement of electric dipoles in adjacent sublattices, exhibit diverse structural phase transition phenomena and distinctive physical properties under the influence of temperature or electric field [5, 7, 8]. The typical reversible antipolar‐polar phase transformation under external electric fields endows AFEs with remarkable variation of polarization, which gives rise to exceptional related performances, including energy storage [9, 10, 11], electrocaloric effect [12, 13, 14, 15], and electrostriction [16, 17], thereby offering application space for energy storage capacitors, solid‐state refrigeration equipment, and electrostriction actuators.

**FIGURE 2:**
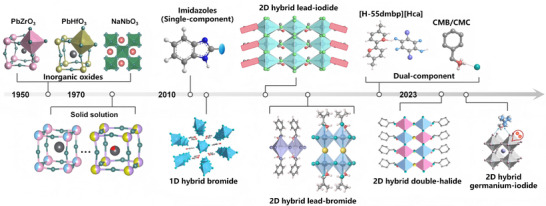
The discovery and major development of antiferroelectrics.

The initial discovery of AFEs could be traced back to the report on the oxide perovskite of PbZrO_3_ in 1951 [18]. Then, the theoretical justification for the existence of antiferroelectricity was declared by Bell Labs [19]. Subsequently, more inorganic oxide antiferroelectrics, including PbHfO_3_ [20] and NaNbO_3_ [21], have been developed. In parallel with the fundamental work, more comprehensive studies on the antiferroelectric solid solutions [22, 23, 24, 25], such as the PbZrO_3_‐PbTiO_3_ solid solution phase diagram, have emerged. These seminal investigations on inorganic oxides characterized primarily by ionic displacements in rigid crystal lattices established a classic paradigm for antiferroelectricity; however, the inherent limitations of these systems, such as structural rigidity and high processing temperatures, have motivated the search for alternative candidates in more tunable and processable systems. As a thriving part of the family of ferroic materials, molecular antiferroelectrics have emerged due to their diverse structures, facile synthesis, and excellent antiferroelectricity. In 2012, Sachio Horiuchi et al. discovered antiferroelectricity in organic benzimidazole molecular crystals, which disclosed that weak intermolecular interactions like hydrogen bonds can also induce robust ordered dipole structures and macroscopic AFE behavior [26]. Subsequently, abundant antiferroelectric physical phenomena have also been observed in emerging hybrid halide perovskites. These findings have enriched the AFE system, which has spurred an expansion of research focus beyond traditional inorganic oxides. In those reported antiferroelectrics, the antiferroelectric mechanisms mainly originate from the order‐to‐disorder motion of dipolar molecules or the ionic displacement (Figure 1b,c). Figure 2 depicts the discovery of antiferroelectrics and the major development of molecular antiferroelectrics during the past decades.

In molecular AFEs, diverse building blocks carrying inherent dipole moments are assembled orderly through their weak intermolecular interactions, including hydrogen bonds and van der Waals forces, generating antiparallel dipole arrangements and AFE order. Compared with traditional inorganic oxide AFEs, molecular counterparts manifest greater structural diversity and tailorability, of which the building units can be precisely modified through molecular design strategies such as functionalization and derivatization, thereby enabling control over the magnitude and orientation of dipoles as well as intermolecular interactions [27, 28]. Meanwhile, the polarization switch of molecular AFEs involves weak interactions; thus, smaller required electric fields are expected in such soft materials, which manifests significant application potential for highly efficient energy storage and low‐consumption refrigeration.

In this review, we categorize molecular antiferroelectrics as well as discuss their structural diversity and the origin of antiferroelectric order. With the breaking of traditional inorganic systems, molecular AFEs yield a broad and versatile platform for boosting the multifunctionality of AFEs. Further, antiferroelectricity‐associated properties, such as the temperature‐ and electric‐field‐induced phase transitions, energy storage properties, and electrocaloric effects, were analyzed. The considerably small required electric fields endow molecular antiferroelectrics with significant application potential for highly efficient and low‐consumption electronic devices. Moreover, we discuss the current challenges and future opportunities in this exciting field.

## 2 Structures of Molecular Antiferroelectrics

### 2.1 Single‐Component Molecular Antiferroelectrics

**FIGURE 3:**
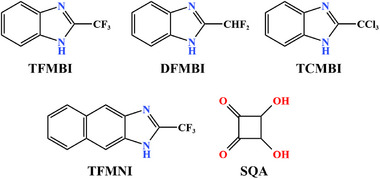
Chemical structures of the reported single‐component molecular antiferroelectrics. Abbreviations are given for clarity.

Single‐component antiferroelectrics are formed by self‐assembling sole organic molecules as basic units through weak intermolecular interactions such as hydrogen bonds and van der Waals forces (Figure 3). A single type of polar molecule adopts an antiparallel arrangement, such as “head‐to‐tail” orientation, resulting in macroscopically compensated dipole moments and a net zero polarization. In such single‐component antiferroelectrics, the polarization switching involves proton motion within the intra‐ and/or intermolecular hydrogen bonds, wherein the cooperative prototropy or proton tautomerism results in the relocation of a hydrogen position and the concurrent interchange of adjacent single and double bonds.

#### 2.1.1 Imidazole

**FIGURE 4:**
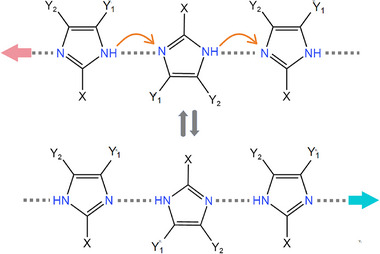
Plarization reversal mechanism through the proton tautomerism of the imidazole moiety.

Imidazole, as a crucial and ubiquitous structural unit in biological systems, manifests excellent chemical stability. A large dipole moment in the imidazoles is almost parallel to the N‐H bond [29, 30]. The proton donor (N─H) and proton acceptor (sp^2^ nitrogen atom) on the imidazole ring can form complementary hydrogen bond pairs, forming a 1D chain‐like supramolecular structure through self‐assembly [26]. Excitingly, the polarity of the chain can be synergistically changed by π bond conversion and proton transfer, which transforms the N─HN bond into the NH─N bond and vice versa (Figure 4). In 2012, Sachio Horiuchi et al. discovered antiferroelectricity above room temperature in benzimidazole‐based organic molecular crystals, including 2‐trifluoromethylbenzimidazole (TFMBI), 2‐difluoromethylbenzimidazole (DFMBI), and 2‐trichloromethylbenzimidazole (TCMBI) [26]. Similar benzimidazole molecules are assembled into a chain‐like structure through hydrogen bonds. These molecular antiferroelectrics show pseudocrystalline plane symmetry perpendicular to the molecular plane when the proton position is ignored. However, in reality, due to the ordered arrangement of protons, the pseudo‐orthogonal lattice (*Pbcm*) of TFMBI and DFMBI crystals is reduced to a monoclinic lattice group (*P*2_1_/*c)*, where the dipolar chains adopt an antiparallel arrangement, leading to antiferroelectric properties along the *a*‐axis. On the contrary, the pseudo‐orthogonal symmetry of the TCMBI crystal is reduced to a polar space group of *P*2, where the antiparallel dipole moment along the transverse molecular axes causes antiferroelectricity along the *a*‐axis, while the permanent polarization is retained along the *b*‐axis by the crystal symmetry. By applying an electric field parallel to the hydrogen‐bonded chain for the three imidazoles, the antiparallel dipoles are coaxially reversed to a parallel state, resulting in an antiferroelectric‐ferroelectric phase transition and typical double hysteresis loops. The values of *P*_max_ of TFMBI, DFMBI, and TCMBI are estimated to be 8, 6, and 8 µC/cm^2^, respectively. This work demonstrates that the hydrogen‐bonded chains self‐assembled from amphoteric molecules are bistable, where the polarization can be electrically switched through proton tautomerism. Later, Kensuke Kobayashi et al. discovered the antiferroelectric properties in a naphthimidazole analogue, 2‐trifluoromethylnaphthimidazole (TFMNI), which forms an antipolar crystal with alternating polarities of the hydrogen‐bonded chains [31]. Typical *P*‐*E* loops can be observed when an electric field is applied to the hydrogen‐bonded chain, where the field‐induced polarization rapidly increases around 30–40 kV/cm and saturates to 8 µC/cm^2^ at ∼50 kV/cm.

#### 2.1.2 Squaric Acid (SQA)

Horiuchi et al. confirmed the antiferroelectricity of the square acid (SQA) [32]. SQA crystallizes in a centro‐symmetric space group *P*2_1_/*m*, where the protons in the hydrogen‐bonded 2D sheets are ordered, leading to the opposite polarization in adjacent sheets. Theoretical calculations suggest that the sublattice polarization reaches up to 11.6 µC/cm^2^, mainly due to the contribution of π bond switching rather than proton displacement. From the *P*‐*E* loops, SQA exhibits extremely high polarization (*P*_m_ ≈ 13.3 µC/cm^2^) and a jump Δ*P* of 10.5 µC/cm^2^ with a minimal hysteresis. Particularly, SQA exhibits a unique polarization switching mechanism, of which the pseudo‐tetragonal symmetry permits the additional 90° rotation of 2D sublattice polarizations in four directions. Note that the microscopic structural changes with cooperative proton transfer for polarization rotations, where the 90° rotation requires transfer of only one proton per molecule while the 180° inversion involves double proton transfer Experiments show that when an electric field is applied in the E*∥*[100] direction, a 90° rotation mainly occurs and induces the FE phase with a polarization of approximately 14 µC/cm^2^, which is in good agreement with theoretical predictions.

### 2.2 Dual‐Component Molecular Antiferroelectrics

In dual‐component molecular antiferroelectrics, organic cations and anions are orderedly arranged by hydrogen bonds, with antiparallel‐arranged dipole moments in adjacent lattice subunits. The antiferroelectric order is constructed through the synergy of ionic displacement and molecular orientation, while the polarization switching is related to the coordinated movement of protons between acid‐base molecules or the rearrangement of molecular orientation.

#### 2.2.1 Cyclohexylammonium Salt

**FIGURE 5:**
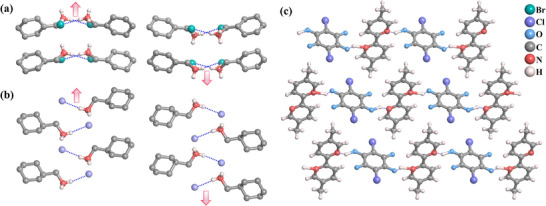
Crystal structure characterizations of reported dual‐component molecular antiferroelectrics, including CMB (cyclohexylmethylammonium bromide, (a), CMC (cyclohexylmethylammonium chloride, (b), and [H‐55dmbp][Hca] (5,50‐dimethyl‐2,20‐bipyridinium chloranilat, (c).

Cyclohexylammonium bromide (CMB) crystallizes in the orthorhombic *Pbca* space group, composed of hydrogen‐bonded dimers. The anion Br^−^ and the cation cyclohexylammonium (CM^+^) are linked by N─H···Br hydrogen bonds, in which the organic CM^+^ cation serves as a dynamic polar unit like a “rotor” to drive the order‐disorder transformation, while halogen anions provide a rigid framework for the system and maintain the stability of the crystal structure. Meanwhile, the halogen ions would effectively modulate the molecular dipole moments and the strength of the hydrogen bond. Cyclohexylamine chloride (CMC) with a more electronegative chlorine atom manifests a similar structure but significantly enhanced intermolecular interactions, as reflected in a shorter hydrogen bond distance of 3.148 Å. The intermolecular interaction plays a crucial role in determining the energy barrier, leading to a higher phase transition temperature for CMC (453 K). Notably, as shown in Figure 5, the arrangement of the two equivalent arrays of the H‐bonding dimers exhibits an opposite arrangement with the same magnitude, which suggests the antipolar feature for AFE. The antiparallel arrangement of electric dipoles in adjacent sublattices leads to a volume multiplication of the unit cell, which is one of the phase transition characteristics of antiferroelectrics. When an electric field is applied along the direction of the antiparallel arrangement of the molecules, two distinct pairs of current peaks and double hysteresis loops can be observed, demonstrating the antiferroelectric order definitively. The values of *P*_max_ of CMB and CMC are 6 and 11.4 µC/cm^2^, respectively, where the difference in polarization magnitude is due to the difference in the strength of intra‐interaction. Meanwhile, both the forward switching field (*E*_A_) from antiferroelectric to ferroelectric and the reverse switching field (*E*_F_) from ferroelectric to antiferroelectric show significant temperature‐dependent behavior, which can be attributed to the reduced energy barrier for the field‐induced phase transition by thermal fluctuations. Similarly, a series of high‐*T*_c_ molecular AFEs of the halogen‐substituted phenethylammonium bromides (*x*‐PEAB, *x* = H/F/Cl/Br) has been developed, renewing the halogen substitution strategy [33].

#### 2.2.2 [H‐55dmbp][Hca] Salt

[H‐55dmbp][Hca] (5,5’‐dimethyl‐2,2’‐bipyridinium chloranilat) belongs to the triclinic *P* $\bar{1}$ space group, where crystallographically independent molecules of 5,5’‐ddimethyl‐2, 2’‐bipyridine and chloranilic acid are alternately hydrogen bonded into a ribbonlike supramolecule parallel to the *b*‐axis (Figure 5c) [34]. The proton ordering is accompanied by a simple unit‐cell doubling of the triclinic cell, as well as the emergence of a dipole moment in each supramolecular ribbon, and the neighboring ribbon in the *c*‐direction has the opposite polarization. From the *P*‐*E* loop, [H‐55dmbp][Hca] crystal exhibited a *P*_max_ of 5 µC/cm^2^. As the temperature rises, the *P*‐*E* curves smear out the polarization jump until the linear *P*‐*E* relationship for normal paraelectrics survives at *T*_c_, which is consistent with the collapse of peaks by broadening in the current–electric field curves.

#### 2.2.3 (H_2_Dabco)BrClO_4_

(H_2_Dabco)BrClO_4_ (H_2_Dabco = N,N’‐1,4‐diazabicyclo[2.2.2]octonium) crystallizes in the orthorhombic *Pn*2_1_ *a*, where the inorganic [ClO_4_] and organic Br···[H_2_Dabco] are stacking layer by layer along the *c*‐axis [35]. As the temperature increases, (H_2_Dabco)BrClO_4_ undergoes equational ferroelectric‐antiferroelectric‐paraelectric phase transitions. For the intermediate‐temperature phase, (H_2_Dabco)BrClO_4_ adopts a higher‐symmetry structure (space group of *P*6_1_22). Microscopically, the oxygen ions in [ClO_4_] ions become disordered with partial occupations on equivalent eight sites. The orientations of Br···[H_2_Dabco] chains remain noncollinear between neighbor layers, that is, with a ∼60° twist around the *c*‐axis. Such a triple structure cancels the net polarization from asymmetric [H_2_Dabco] groups, by forming a rare screw antiferroelectric order of local dipoles.

### 2.3 Hybrid Molecular Antiferroelectrics

The development of multi‐component molecular antiferroelectrics is mainly focused on organic‐inorganic hybrid halide perovskites, derived from the conventional inorganic oxide perovskites. The organic components and the inorganic framework are synergistically coupled through hydrogen bonds, van der Waals forces, and coordination bonds [36]. When the volume of the organic ammonium exceeds the tolerance factor limit, the original 3D continuous octahedral inorganic framework undergoes dimensionality reduction to form low‐dimensional hybrid perovskite structures, such as 2D layered, 1D chain, or 0D isolated clusters, where the structural confinement effect provides a favorable crystallographic environment for generating ferroelectric order [37, 38]. From the perspective of microstructure, the distorted inorganic skeleton can induce a large polarization, while the flexible organic ammoniums undergoing the ordered‐disordered transformation induce the ferroelectric phase transition. Excitingly, through molecular engineering, such hybrid materials can also achieve dipole antiparallel alignment and excellent antiferroelectric properties. The dynamic movement or orientation of organic cations can be easily regulated by external fields, such as temperature or electric field, which provides an effective driving mechanism for structural phase transitions. The hybrid perovskite molecular antiferroelectrics are mainly 2D and 1D.

#### 2.3.1 2D Hybrid Perovskite Molecular Antiferroelectrics

**FIGURE 6:**
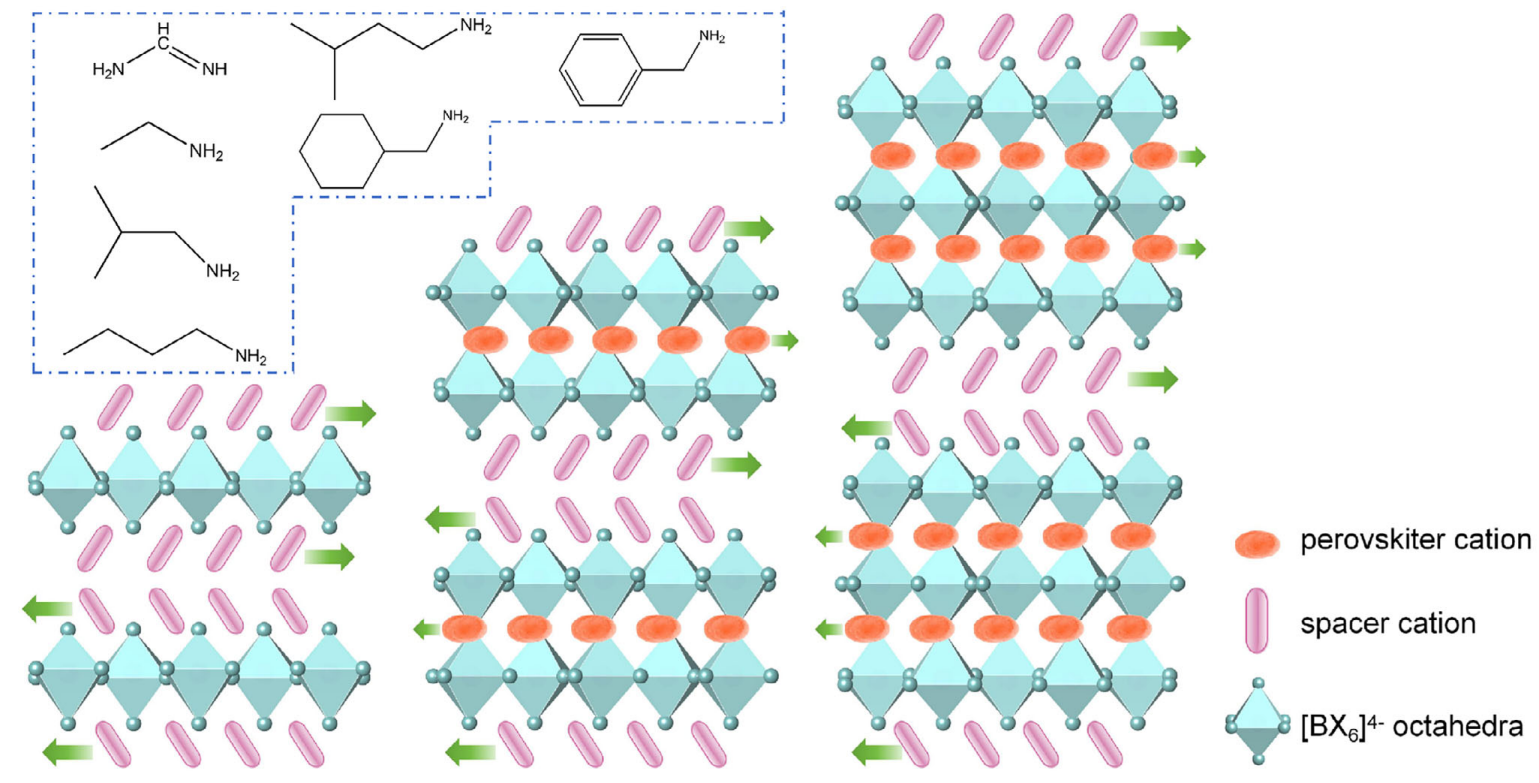
Crystal structure characterizations of 2D hybrid perovskite molecular antiferroelectrics. The inset shows organic ammonium as a flexible unit for constructing 2D hybrid perovskite molecular antiferroelectrics.

Dimension reduction through “chemical embedding” is a common approach for developing 2D hybrid perovskite molecular ferroelectrics/antiferroelectrics. The general formula for a 2D hybrid perovskite is A'_m_A_n‐1_B_n_X_3n+1_ (where A' is an spacer cation; A is a perovskitizer cation such as Cs^+^; B is a metal ion including Pb^2+^, Ge^2+^; X is a halogen anion, such as Cl^−^, Br^−^, and I^−^; n is the number of layers of the inorganic skeleton), as shown in the Figure 6. The organic spacer cations are connected to the inorganic framework through hydrogen bonds, and the perovskitizer cations are confined in the cavities constructed by the corner‐sharing octahedra, while the inorganic framework provides a confined space for the cations to arrange orderly. Consequently, the dynamic motion of cations gives rise to antiparallel alignment of adjacent dipoles, resulting in macroscopic zero net spontaneous polarization and generating antiferroelectric order. The flexible and variable ordered‐disordered transformation of the organic cations and the varying degree of distortion within the inorganic framework synergistically drive the phase transition.

**FIGURE 7:**
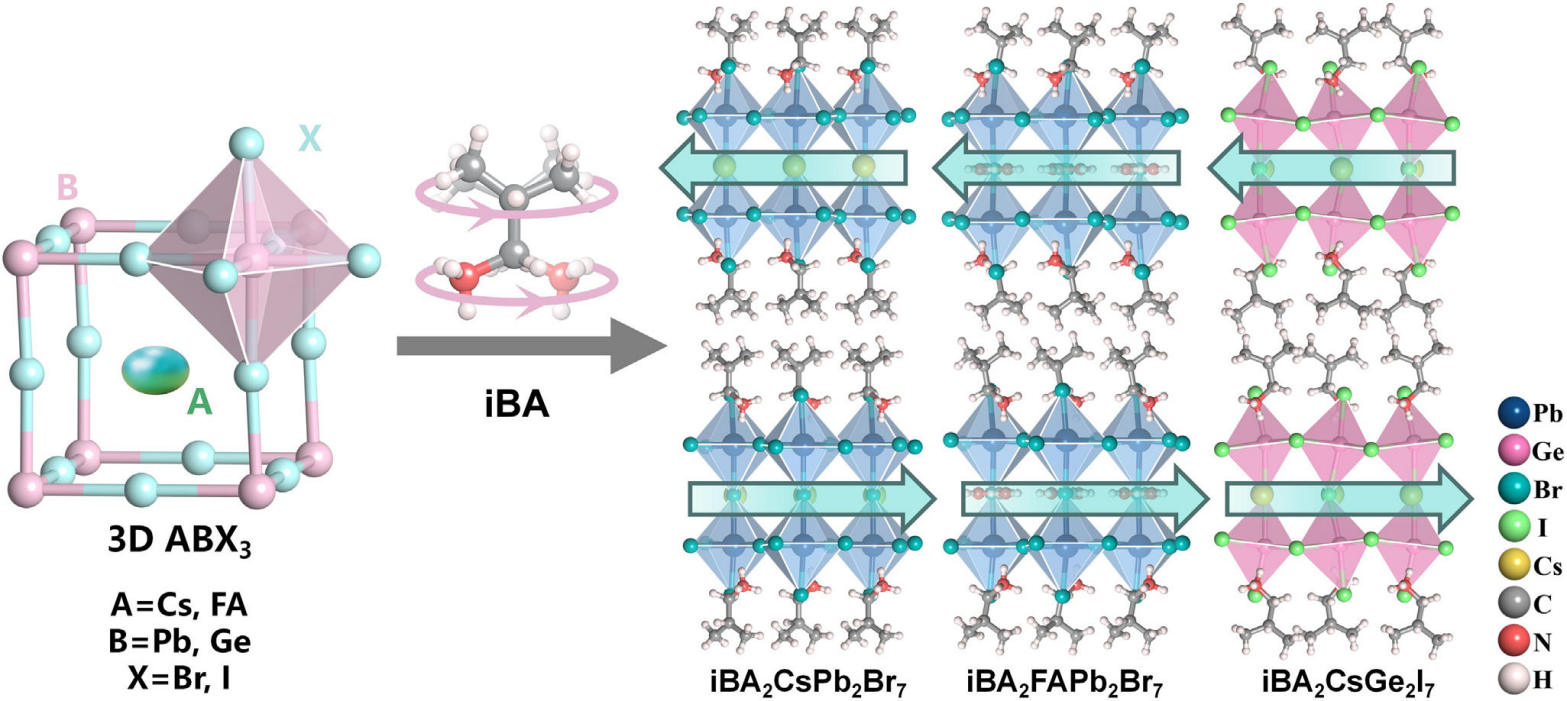
Alloying organic iBA^+^ into 3D ABX_3_ to construct 2D hybrid perovskite molecule antiferroelectrics.

The organic ammonium, iso‐butylammonium, has been regarded as a flexible unit to construct hybrid perovskite molecular antiferroelectrics (Figure 7). For example, by alloying iso‐butylammonium into the cubic 3D CsPbBr_3_, a new case of 2D hybrid perovskite molecular antiferroelectrics, (i‐BA)_2_CsPb_2_Br_7_ has been developed, which crystallizes in the orthorhombic space group of *Pmnb* [39]. As Figure 7 depicts, (i‐BA)_2_CsPb_2_Br_7_ possesses a typical 2D sandwich‐like architecture with i‐BA^+^ cations separating inorganic layers and Cs^+^ confined in the cavities constructed by the corner‐sharing [PbBr_6_]^4−^ octahedra. It is considerably interesting that the combination of the dynamic movement of Cs^+^ and i‐BA leads to the antiparallel alignment of adjacent dipoles and antiferroelectric order along its *b*‐axis. From the single‐crystal structural analyses, the chain length and spatial volume of the spacer cations play a key role in the crystal configuration of the hybrid perovskite. The smaller interlayer spacing between adjacent inorganic perovskite layers indicates a stronger spatial confinement effect and greater spatial repulsion between adjacent parallel dipoles [40]. Specifically, the average spacing distance between adjacent organic layers in isobutylammonium‐based hybrid perovskites is significantly smaller than that in n‐butylammonium‐based hybrid perovskites, indicating a stronger geometric constraint effect. As a result, the antipolar arrangement within the perovskite structure is also affected by spatial repulsion, which contributes to the stable presence of the antipolar arrangement in the antiferroelectric lattice structure. These findings confirm that the branched isobutylammonium is a functional unit for constructing hybrid perovskite molecule antiferroelectrics, which unveils a pragmatic, sophisticated pathway for the precise assembly of antiferroelectric ordered architectures. Further, the antiferroelectric properties of hybrid perovskite molecule antiferroelectrics were optimized by enhancing the stereo‐activity of lone‐pair electrons of the metal [41]. Structural analysis and electron local function calculations suggest that the stereochemical expression of lone‐pair electrons in Ge shows a significant repulsive effect on the bonding electrons, promoting an eccentric displacement of Ge and obvious antiferroelectric characteristics. (i‐BA)_2_CsGe_2_I_7_ shows well‐defined double *P*‐*E* hysteresis loops over a wide temperature range with a giant maximum polarization up to 18.8 µC cm^−2^, achieving a new high record among molecular antiferroelectrics.

**FIGURE 8:**
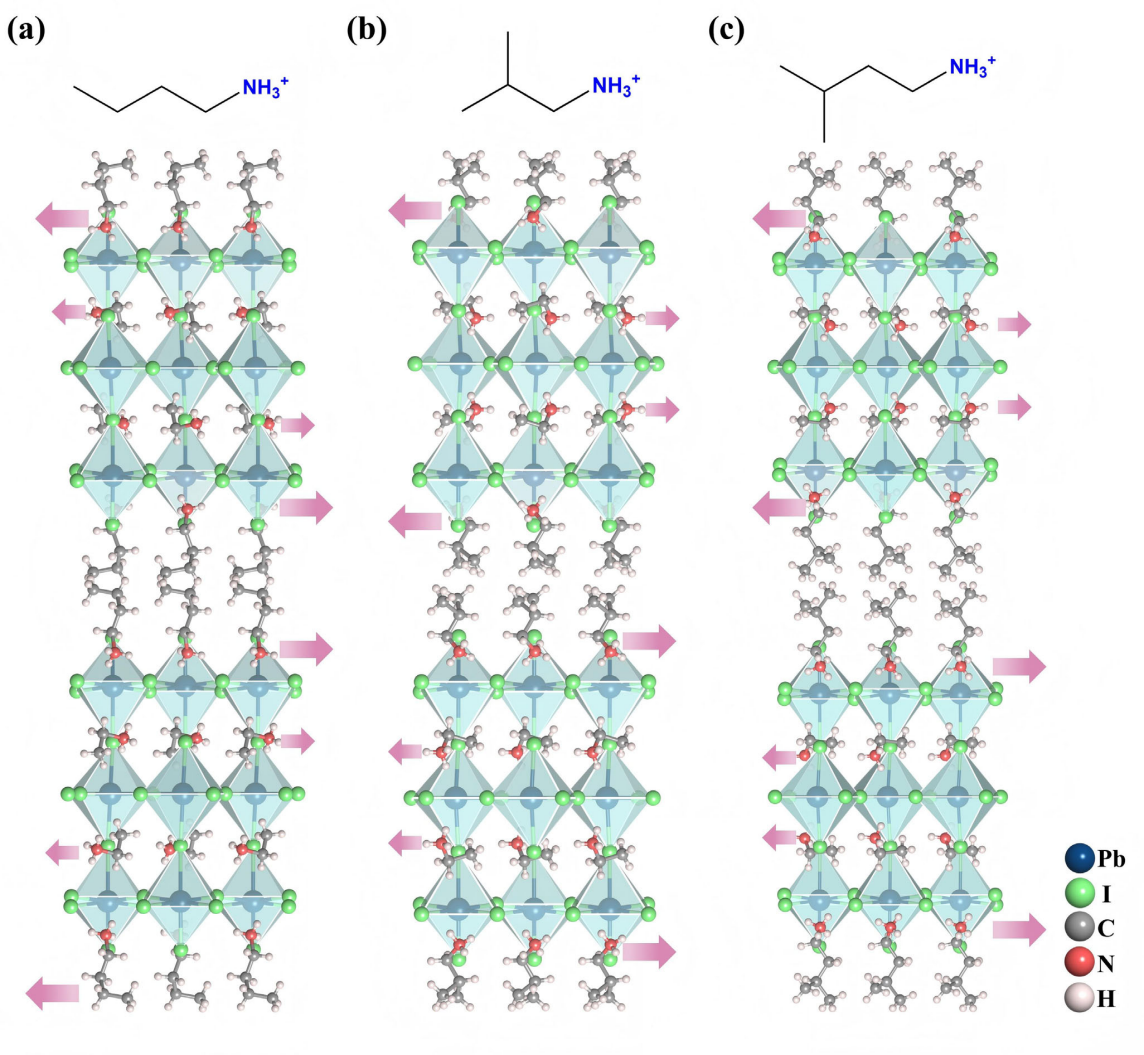
Crystal structure characterizations of (n‐BA)_2_(EA)_2_Pb_3_I_10_ (a); (iBA)_2_(EA)_2_Pb_3_I_10_ (b); (i‐AA)_2_(EA)_2_Pb_3_I_10_ (c).

Ethylammonium (EA) has been used to construct a series of 2D halide perovskite antiferroelectrics, serving as a “cage‐confined rotator” triggering polarization and phase transitions. In particular, the soft [PbI_6_]^4−^ inorganic framework provides a flexible and geometrically confined nanoenvironment for organic ammines to adopt an antiparallel arrangement [42, 43, 44]. Meanwhile, the ethylammonium moieties impose a highly anisotropic strain on the inorganic framework via N─H···I hydrogen bonds, generating a specific and energy‐minimizing distortion pattern of the soft [PbI_6_]^4−^ network. Figure 8 shows the crystal structure characterizations of the 2D lead‐iodide perovskite molecule antiferroelectrics, of which ethylammonium serves as “perovskitizer”.

Through incorporating different flexible functional units into a 3D template, a series of 2D hybrid perovskite antiferroelectrics has been developed. By alloying a flexible organic cation, isopentylammonium (i‐AA^+^), a new soft antiferroelectric, (i‐AA)_2_CsPb_2_Br_7_, has been developed, of which the cooperativity between reorientation ordering of the i‐AA^+^ ammoniums and the ionic shift of the Cs^+^ cation induces its multiple ferroelectric‐antiferroelectric‐paraelectric phase transitions [45]. Beyond the substitution of organic moieties, there is growing emphasis on optimizing inorganic frameworks, driven by the pursuit of environmentally friendly materials. Particularly, 2D double halide perovskites display infinite structure compatibility and tunability, allowing rational incorporation of organic cations into the perovskite frameworks, which could be reoriented under electric field control. By incorporating flexible cyclic organic cyclohexylammonium, CHMA^+^, into 3D organic double Cs_2_AgBiBr_6_, a newly environmentally friendly 2D double perovskite antiferroelectric, (CHMA)_2_CsAgBiBr_7_, has been successfully designed with a *P*_max_ of 4.2 µC/cm^2^ [46]. Similar to the Pb‐based counterparts, the inorganic scaffolds composed of the distorted alternating [AgBr_6_]^5−^ octahedra and [BiBr_6_]^3−^ octahedra with Cs^+^ cations deviate from the center of the cavity, whereas bilayers of organic CHMA^+^ cations separate the inorganic slabs. It is noteworthy that the protonated NH_3_^+^ groups of CHMA^+^ cations form N─H∙∙∙Br hydrogen‐bonds to the terminal Br atoms of inorganic layers, and align in the opposite direction. Consequently, the antiparallel arrangement of CHMA^+^, slight displacements of Cs^+^, and a distorted inorganic framework jointly result in the complete elimination of net polarization in the unit cell and zero bulk polarization.

It is worth noting that such 2D FE/AFE crystals offer complementary merits, including ultrathin geometry, free of dangling bonds, structure flexibility, and easy integration with other materials. In 2023, the crystal structure and electrical properties of the 2D monolayered hybrid perovskite (benzylammonium)_2_PbBr_4_ were studied, consisting of staggered layers of corner‐sharing [PbBr_6_]^4−^ octahedra frameworks interleaved by bilayer organic PMA cations. The surface morphology of the single crystal shows a series of terraces with a height of ≈1.6 nm, indicating the 2D nature of (benzylammonium)_2_PbBr_4_ [47]. Above‐room temperature, (benzylammonium)_2_PbBr_4_ manifests AFE order with a considerable *P*_max_ of ≈7.6 µC cm^−2^ and an unprecedented *E*_max_ of ≈1000 kV cm^−1^. The reorientation of the PMA molecules in the two sublayers results in the cancellation of electric dipoles from the organic part, while the [PbBr_6_]^4−^ octahedron endures no distortion with the Pb ion sitting in the center of the octahedra, synergistically leading to a nonpolar phase with zero net polarization.

#### 2.3.2 1D Hybrid Perovskite Molecular Antiferroelectrics

**FIGURE 9:**
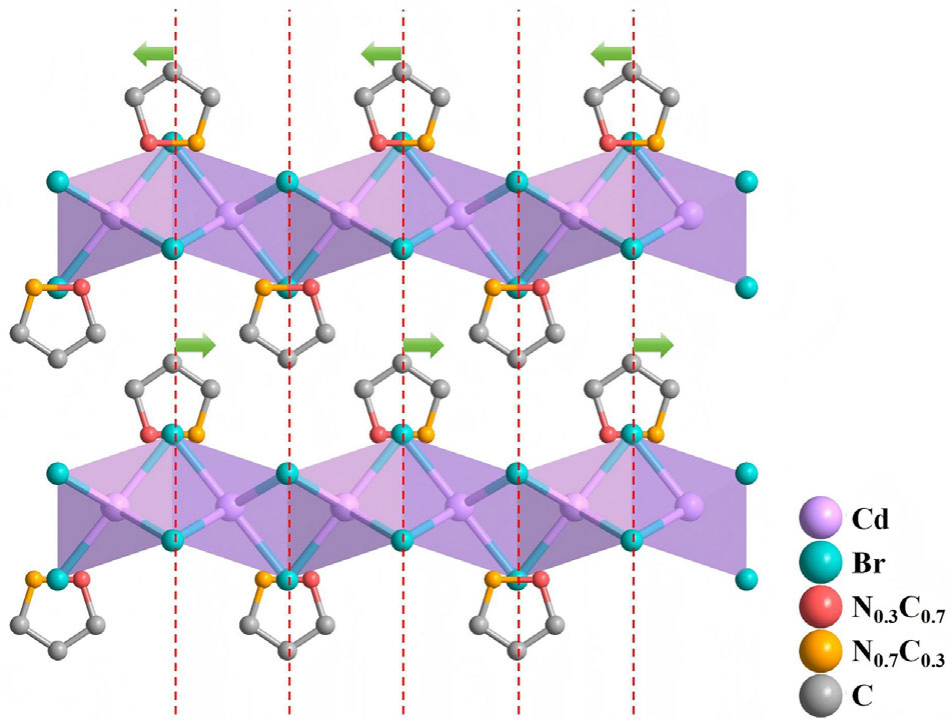
Crystal structure characterizations of (3‐pyrrolinium)CdBr_3_.

A new compound (3‐pyrrolinium)CdBr_3_ with a striking AFE characteristic revealed by clear double *P*‐*E* hysteresis loops has been developed. The antiferroelectric phase of (3‐pyrrolinium)CdBr_3_ was assumed as *C*2/*m*11 [48]. The modeled 3‐pyrrolinium cation shows disordered, where the ratios of N and C for the two atoms bonded by the double bond are 0.7:0.3 and 0.3:0.7. As shown in Figure 9, such a model indicates that there are antiparallel electric dipole moments along the *c*‐axis. A characteristic double hysteresis *P*‐*E* loops shows one distinct feature of the antiferroelectrics, where the forward phase switching field and the backward switching field can be estimated by extrapolating the two steepest sections of the hysteresis loop and obtaining their intersections with the horizontal axis. Similarly, noticeable antiferroelectric behavior has been demonstrated in (4‐methylpiperidium)CdCl_3_, of which the modeled cations adopting a 2‐fold orientationally disordered state with an unequal ratio of molecular orientation distribution exhibit antiparallel alignment [49]. Excitingly, compared to previously reported (3‐pyrrolinium)CdBr_3_, cationic customization and halogen engineering bring about a significant improvement of Curie temperature (Δ*T*_c_ = 114.4 K) to reach 361 K of (4‐methylpiperidium)CdCl_3_.

To sum up, the emerging molecular antiferroelectrics offer a compelling combination of properties, which not only retain the core functional characteristics of traditional inorganic antiferroelectrics but also introduce the distinct advantages of molecular materials. Notably, their structural flexibility allows for tunable performance through the regulation of compositional building blocks, enabling excellent antiferroelectric properties. Table 1 summarizes the reported molecular antiferroelectrics with their antiferroelectricity‐related properties.

**TABLE 1:** Summary of reported molecular antiferroelectrics with their antiferroelectricity‐related properties.

Classification	Compound	Phase transition	*P*_max_ (µC/cm^2^)	*T*_c_ (K)	*E*_FE‐AFE_, *E*_AFE‐FE_ (kV/cm)	Refs.
Single‐component	TFMBI	AFE(*P*2_1_/*c*)	8	—	5, 13	[26]
	DFMBI	AFE(*P*2_1_/*c*)	6	—	50, 60	[26]
	TCMBI	AFE(*P*2_1_)	8	—	30, 50	[26]
	TFMNI	AFE(*P*2_1_/*n*)	7.5	—	10, 25	[31]
	SQA	AFE(*P*2_1_/*m*)→PE(*I*4/*m*)	10.5	373	125, 120	[32]
Dual‐component	[H‐55dmbp][Hca]	AFE(*P* $\bar{1}$)→PE(*P* $\bar{1}$)	5	318	140, 150	[32]
	CMB	AFE(*Pbca*)→FE(*P*4*mm*)→PE(*P*4/*nmm*)	6	368	7.8, 44.9	[50]
	CMC	AFE(*Pnma*)→PE(*P*4/*nmm*)	11.4	453	2, 49	[51]
	F‐PEB	FE(*P*2_1_)→AFE(*P*2_1_/*m*)→PE(*Pmma*)	3.2	374	34, 25	[33]
	(H_2_Dabco)BrClO_4_	FE(*Pn*2_1_ *a*)→AFE(*P*6_1_22)→PE(*P*6_3_ */mmc*)	0.5	490	4, 8	[35]
Hybrid	(3‐pyrrolinium)CdBr_3_	FE(*Cmc*2_1_)*→*AFE(*C*2*/m*)*→PE(Cmcm)*	7.0	244	1.9, 7.2	[39]
	(4‐MePD)CdCl_3_	FE(*Pna*2_1_)*→*AFE*→PE(Pnam)*	1.49	361	4.7, 11.3	[49]
	(i‐BA)_2_(FA)Pb_2_Br_7_	AFE(*Pnma*)*→PE(I*4*/mmm)*	3.7	303	41, 54	[40]
	(i‐AA)_2_(EA)_2_Pb_3_I_10_	FE(*Cmc*2_1_)*→*AFE(*Pmcn*)*→PE(I*4*/mmm)*	5.2	340	10.6, 21.4	[42]
	(i‐BA)_2_(EA)_2_Pb_3_I_10_	FE(*Pmn*2_1_)*→*AFE(*Pmcn*)*→PE(I*4*/mmm)*	5.0	341	1.5, 11.8	[43]
	(n‐BA)_2_(EA)_2_Pb_3_I_10_	FE(*Cmc*2_1_)*→*AFE(*Pbca*)*→*PE*(I*4*/mmm)*	5.6	363	10, 32	[44]
	(i‐BA)_2_CsPb_2_Br_7_	AFE(*Pmnb*)*→*PE(*Cmca*)	6.3	353	40, 75	[40]
	(i‐AA)_2_CsPb_2_Br_7_	FE(*Cmc*2_1_)*→*AFE(*Pbcm*)*→*PE(*I*4/*mmm*)	5.8	346	1.1, 5.2	[45]
	(i‐BA)_2_CsGe_2_I_7_	AFE(*Pmmn*)*→*PE(*Fmmm*)	18.8	403	15.09, 6.98	[41]
	(PMA)_2_PbBr_4_	FE(*Pca*2_1_ */Cmc*2_1_)*→*AFE(*Pbca*)*→*PE	6.7	402	100, 400	[47]
	(CHMA)_2_CsAgBiBr_7_	FE(*Ama*2)*→*AFE(*Cmcm*)*→*PE(*I*4/*mmm*)	4.2	378	6.6, 135	[46]

SQA = Squaric acid; [H‐55dmbp][Hca] = 5,50‐dimethyl‐2,20‐bipyridinium chloranilate; F‐PEB = F‐phenethylammonium bromide; CMB = Cyclohexylmethylammonium bromide; CMC = Cyclohexylmethylammonium chloride; TFMBI = 2‐trifluoromethylbenzimidazole; DFMBI = 2‐difluoromethylbenzimidazole; TCMBI = 2‐trichloromethylbenzimidazole; TFMNI = 2‐trifluoromethylnaphthimidazole; H_2_Dabco = N’‐1,4diazabicyclo[2.2.2]octonium; 4‐MePD = 4‐methylpiperidium; i‐BA = iso‐butylammonium; i‐AA = iso‐amylammonium; EA = ethylammonium; n‐BA = n‐butylammonium; PMA = benzylammonium; CHMA = cyclohexylmethylammonium.

## 3 Properties

### 3.1 Temperature‐Induced Structural Phase Transitions

Molecular antiferroelectric materials manifest structurally flexible, in which the phase transition behavior often involves “ordered‐disordered” transitions of flexible organic units. In the antiferroelectric phase, molecular units are highly ordered by intermolecular or intramolecular hydrogen bonds, where the dipole moments in adjacent sublattices are antiparallel. As the temperature rises into the paraelectric phase, the thermal motion of the molecules intensifies, and the organic units undergo conformational disordering, leading to a highly symmetrical crystal structure.

**FIGURE 10:**
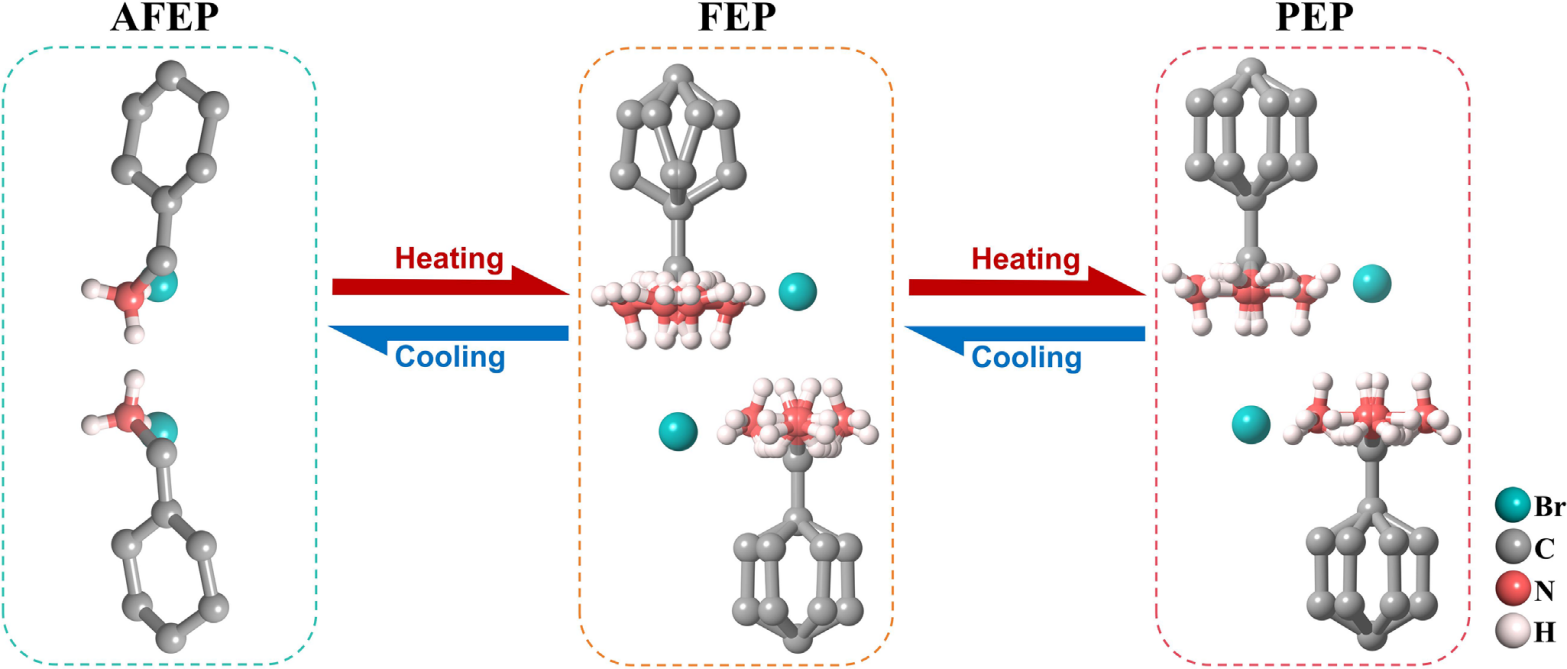
Crystal structures of CMB at different phases.

For example, in the dual‐component molecular antiferroelectrics, cyclohexylammonium bromide, the flexible cyclohexylammonium (CM) undergoes a transformation from structurally ordered to partially disordered to highly disordered, inducing a structural phase transition of antiferroelectric‐ferroelectric‐paraelectric [48]. At the antiferroelectric phase (AFEP), CMB crystallized in a centrosymmetric space group of *Pbca*, where the cyclohexylammonium cations arrange orderly, connected with Br^−^ through hydrogen bonds, forming a dimer structure with antiparallel arrangement. As expected, at ferroelectric phase (FEP), CMB crystallized in a polar tetragonal space group of *P*4*mm*, where the NH_3_^+^ groups become partially rotationally disordered. Note that there are two different geometries for the H‐bonding dimers in the unit cell (I, II), of which the donor‐acceptor distances show differences. The ionic shift relative to the bromide ion is different‐sized (*Δd*_1_≠*Δd*_2_), contributing a lot to the ferroelectric polarization. As the temperature rises further, CMB in paraelectric phase (PEP) possesses the center inversion symmetry, where the cyclohexylammonium is highly disordered, satisfying the fourth rotational symmetry and occupying eight equivalent positions. As a consequence, the origin of electric polarization can be ascribed to the reorientation of organic cations and relative displacement of anionic moieties, while the ordering‐disordering transformation of organic components drives the occurrence of the FEP‐AFEP‐PEP phase transition (Figure 10).

**FIGURE 11:**
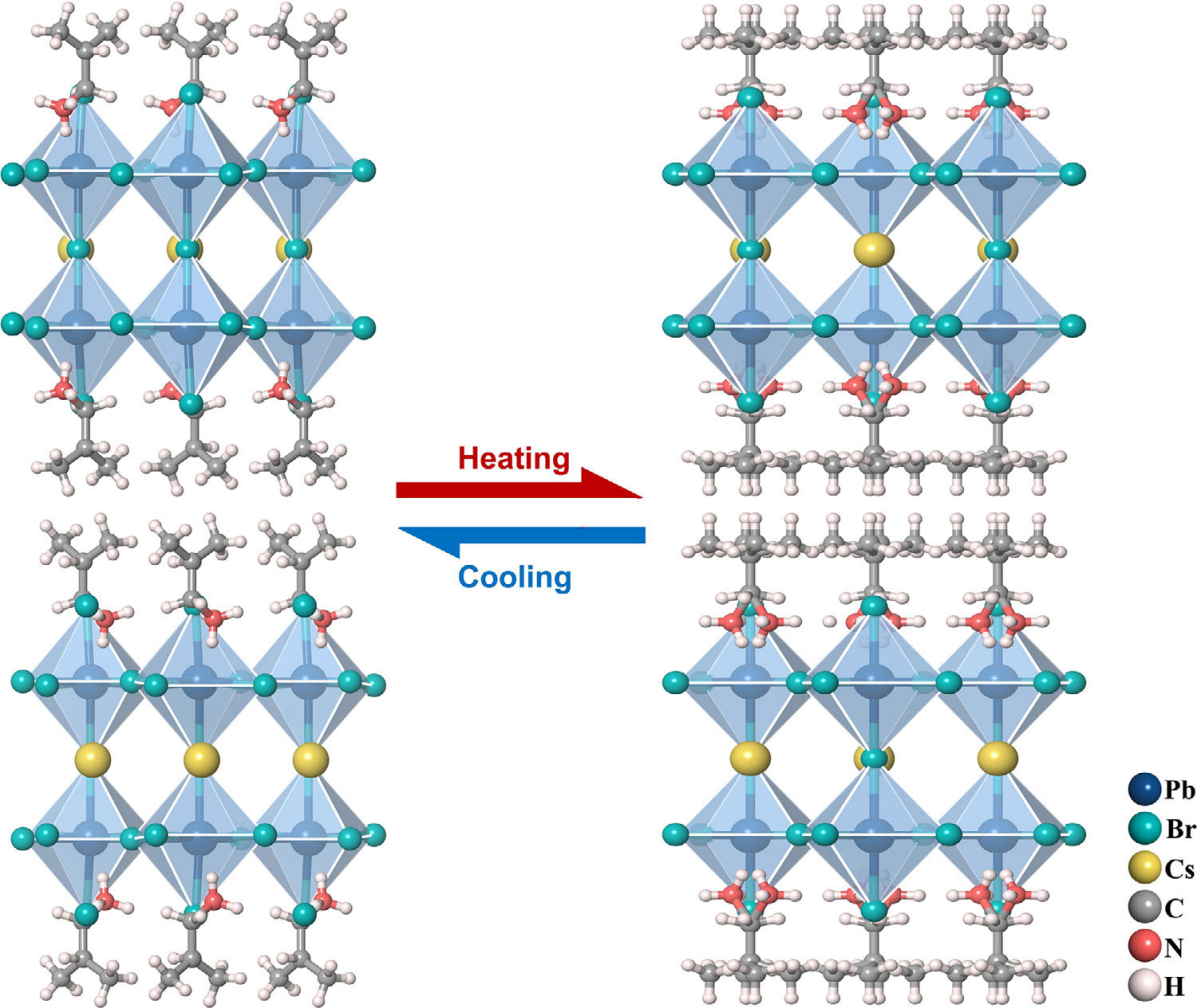
Crystal structures of (i‐BA)_2_CsPb_2_Br_7_ at different phases.

**FIGURE 12:**
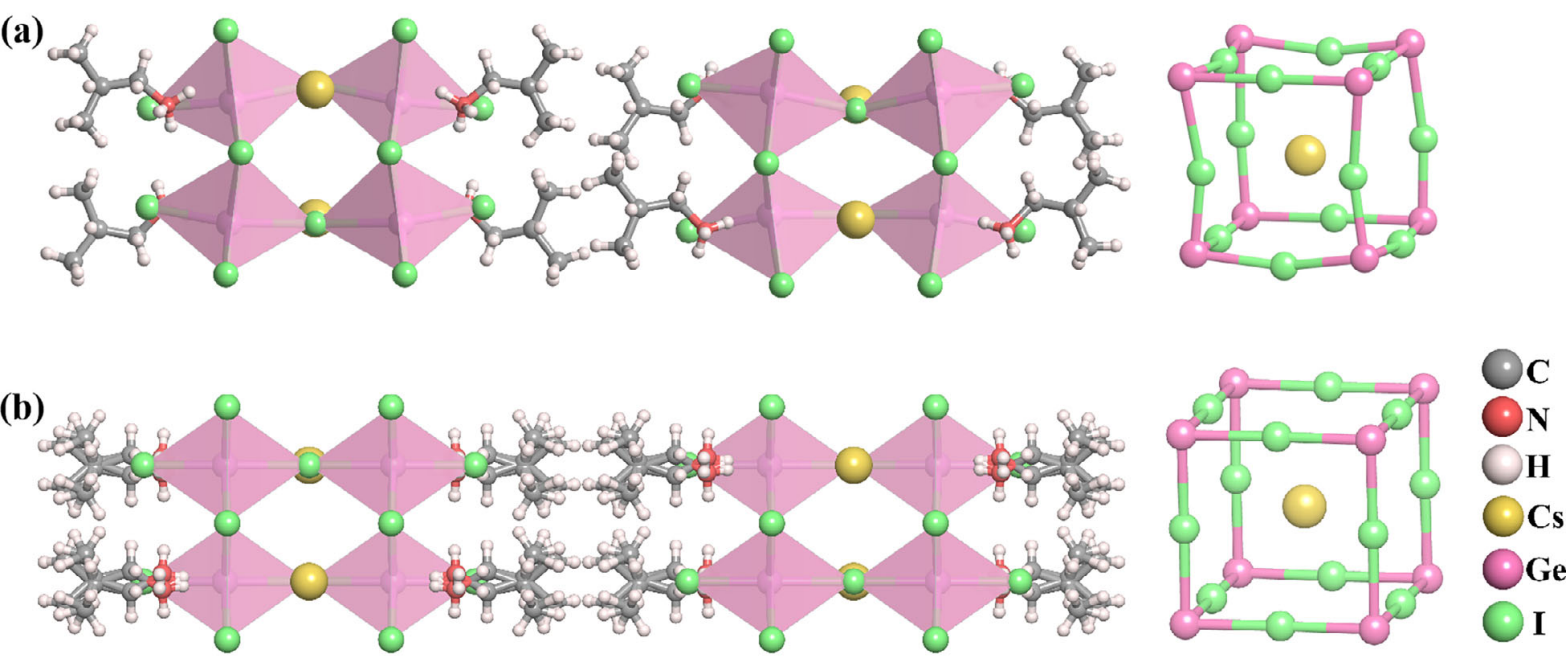
Crystal structures and inorganic framework of (i‐BA)_2_CsPb_2_Br_7_ at antiferroelectric phase (a) and paraelectric phase(b).

The phase transitions of low‐dimensional molecular‐based hybrid antiferroelectrics involve the order‐disorder transformation of organic cations, as well as changes in the degree of distortion and displacement within the inorganic framework. Take (i‐BA)_2_CsPb_2_Br_7_ as a typical example [39]. At the antiferroelectric phase, (i‐BA)_2_CsPb_2_Br_7_ crystallized in the orthorhombic space group of *Pmnb*. Cs^+^ atoms confined in the cavities constructed by the corner‐sharing [PbBr_6_]^4−^ octahedra and i‐BA^+^ ammoniums in two adjacent layers exhibit a distinct and antiparallel shift, which generates antiferroelectric order. As the temperature increases, (i‐BA)_2_CsPb_2_Br_7_ undergoes a reversible phase transition, revealed by the peak‐like dielectric anomaly and exothermic/endothermic peaks in differential scanning calorimetry (DSC) curves. At the paraelectric phase, (i‐BA)_2_CsPb_2_Br_7_ still crystallizes in the orthorhombic centrosymmetric space group *Cmca*, where the distortion of the octahedron significantly decreased, and Cs atoms are located in the middle of cavities (Figure 11). Meanwhile, the organic cations become a highly disordered state at high temperature, satisfying the demands of crystallographic symmetry. Therefore, thermally triggered order‐disorder dynamic transformation of organic moieties and displacement of inorganic Cs atoms synergistically lead to above‐room‐temperature antiferroelectric‐paraelectric phase transition behavior of (i‐BA)_2_CsPb_2_Br_7_. Stereochemically active expression of lone‐pair electrons in metal ions is also one of the key factors in regulating phase transition behavior [41]. For example, at the antiferroelectric phase of (i‐BA)_2_CsGe_2_I_7_, the stereochemical activity expression of the lone pair of electrons induces significant structural distortion in the inorganic skeleton, where the eccentric and antiparallel displacement contributes a lot to the antiferroelectric polarization. When the temperature rises, the stereochemical activity of the lone‐pair electrons decreases significantly, thereby reducing the octahedral distortion and eliminating antiferroelectric polarization (Figure 12).

**FIGURE 13:**
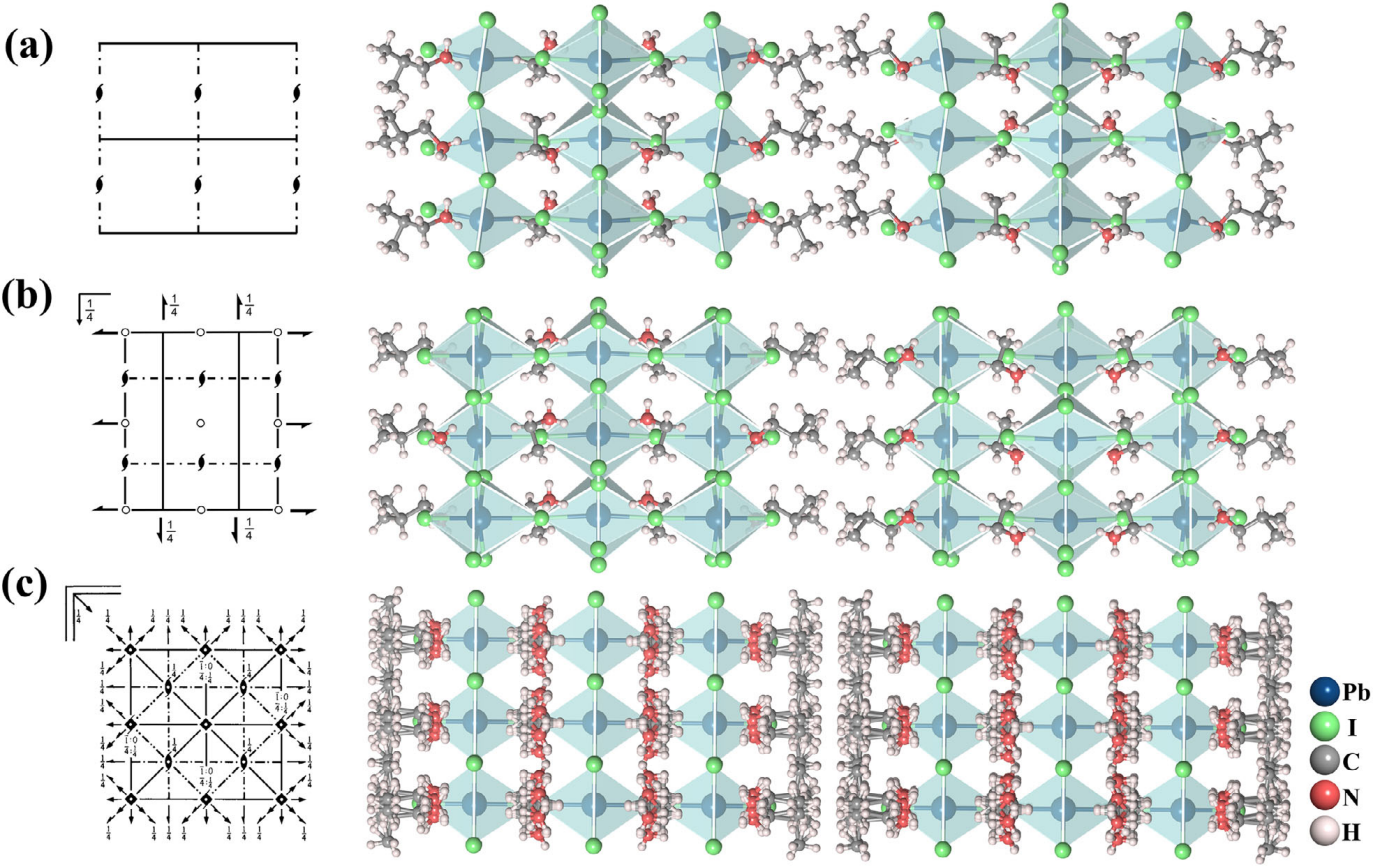
Crystal structures of (iBA)_2_(EA)_2_Pb_3_I_10_ at ferroelectric (a), antiferroelectric (b), and paraelectric phase(c).

Due to the different dynamic behaviors of cations, the compounds may undergo more complex phase transitions, such as ferroelectric‐antiferroelectric‐paraelectric consecutive phase transition [42, 43, 44]. As shown in Figure 13, at FEP, (i‐BA)_2_(EA)_2_Pb_3_I_10_ crystallizes in the *Pmn*2_1_ space group, consisting of slightly twisted [Pb_3_I_10_]_∞_ inorganic layers and organic ammonium tightly attached to the [PbI_6_]^4−^ octahedron [43]. Note that the bulky i‐BA^+^ moieties exhibit the same orientation, generating a parallel arrangement of dipoles and ferroelectric polarization. At AFEP (322 K), (i‐BA)_2_(EA)_2_Pb_3_I_10_ crystallizes in the *Pmcn* space group, with the i‐BA^+^ and EA^+^ cations in adjacent layers present an antiparallel arrangement and generate macroscopical antiferroelectric order. As the temperature increases, transitioning into the paraelectric phase, (i‐BA)_2_(EA)_2_Pb_3_I_10_ transforms into the tetragonal *I*4/*mmm* space group. The i‐BA^+^ and EA^+^ cations become significantly disordered with eight disordered orientational populations, while the inorganic [PbI_6_]^4−^ octahedra present a highly symmetrical configuration, and the central symmetrical packing causes macroscopic polarization to disappear. Such centrosymmetric packing of (i‐BA)_2_(EA)_2_Pb_3_I_10_ eliminates the macroscopic polarization at PEP.

### 3.2 Electric Field ‐Induced Structural Phase Transitions in Molecular Antiferroelectrics

Under a sufficiently strong applied electric field, antiferroelectric bodies undergo a field‐induced phase transition from AFEP to FEP. The microscopic essence of this transformation lies in the fact that the electric field overcomes the anti‐parallel coupling effect of dipoles between adjacent sublattices, forcing the dipoles to flip in coordination and form a macroscopic net polarization.

#### 3.2.1 TFMNI

**FIGURE 14:**
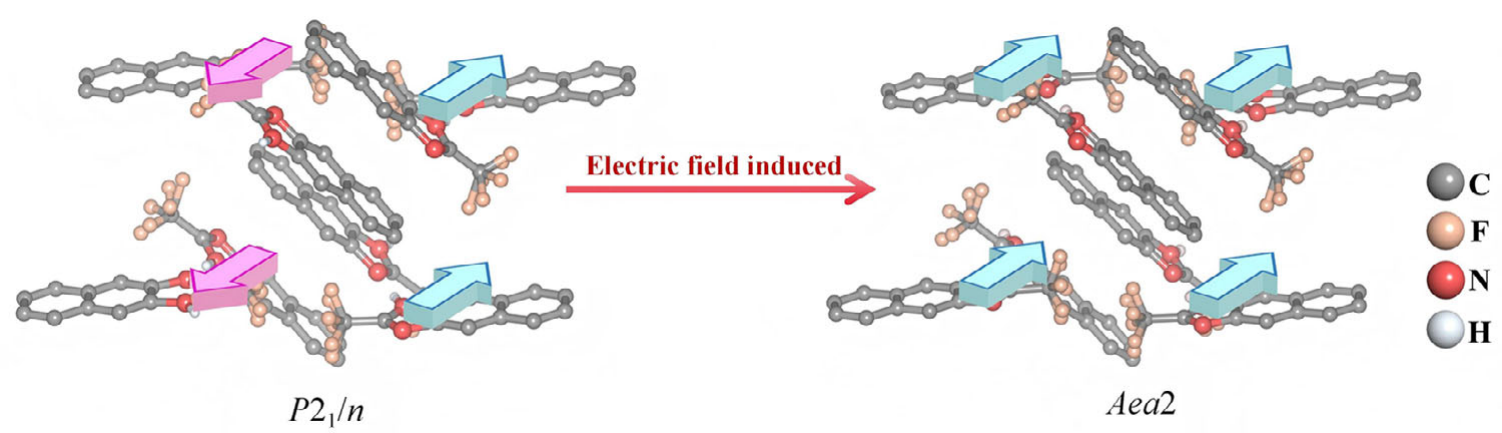
Electric‐field‐induced structural phase transitions in TFMNI.

Kensuke Kobayashi et al. studied the field‐induced phase transition from AFEP to FEP of 2‐Trifluoromethylnaphthimidazole (TFMNI) under the electric field [31]. At zero field, TFMNI crystallized in the monoclinic space group *P*2_1_/*n*, of which the lattice exhibits pseudo‐orthogonal symmetry and forms ferroelastic twins. The hydrogen‐bonded molecules construct linear chains along the [101] direction of the crystal. Under an external electric field of 40 kV/cm, the crystal undergoes obvious detwinning, accompanied by a structural phase transition from the monoclinic phase to the orthorhombic phase (space group *Aea*2) (Figure 14). It is worth noting that the dipole direction of the hydrogen bond chain changes from anti‐parallel to parallel alignment.

#### 3.2.2 (i‐BA)_2_(EA)_2_Pb_3_I_10_

**FIGURE 15:**
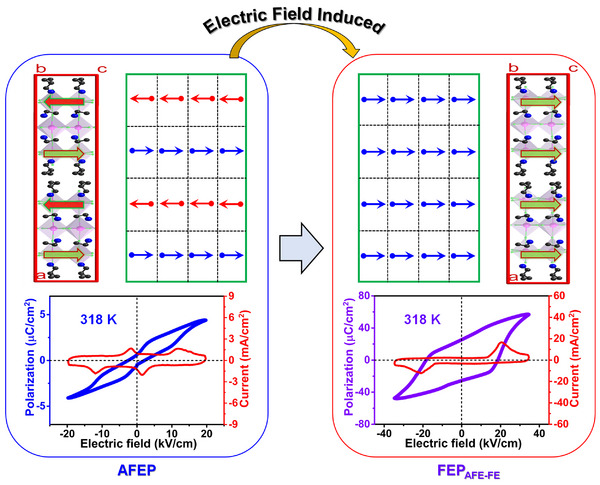
Graphical model of dipoles of (i‐BA)_2_(EA)_2_Pb_3_I_10_ at AFEP and electric‐field induced ferroelectric phase FEP_AFE‑FE_. The arrowheads denote the direction of dipoles. Reproduced with permission [43]. Copyright 2024, American Chemical Society.

The field‐induced antiferroelectric‐ferroelectric phase transition (i‐BA)_2_(EA)_2_Pb_3_I_10_ (i‐BA = isobutylammonium, EA = ethylammonium) has been investigated [43]. At AFEP, under a sufficiently large external electric field applied along the *c*‐axis of the crystal, the two pairs of current peaks in the current‐electric field curve become one pair, accompanied by the change of the double *P*‐*E* loop to a single *P‐E* ferroelectric loop. The polarization reaches up to 30 µC cm^−2^, slightly higher than that of the classical ceramic ferroelectric BaTiO_3_ and far superior to that of most known molecular ferroelectrics [52, 53]. As shown in Figure 15, a speculative model was constructed to disclose such an antiferroelectric‐ferroelectric structural transition. At AFEP, the dipoles of adjacent sublattices are antiparallel, while the antiparallel dipoles are forced to become parallel. Structurally, such a field‐induced transition can be attributed to the reorientation of organic cations. From the theoretical calculations and structural symmetry analysis, the field‐induced ferroelectric phase FEP_AFE‐FE_ crystallized in the monoclinic space group of *Bm*. Half of the EA and i‐BA cations have achieved dipole inversion by rotating 180° around the *a*‐axis, and the barrier of the antiferroelectric‐ferroelectric phase transition is approximately 1.16 eV.

### 3.3 Electrostrictive Properties

**FIGURE 16:**
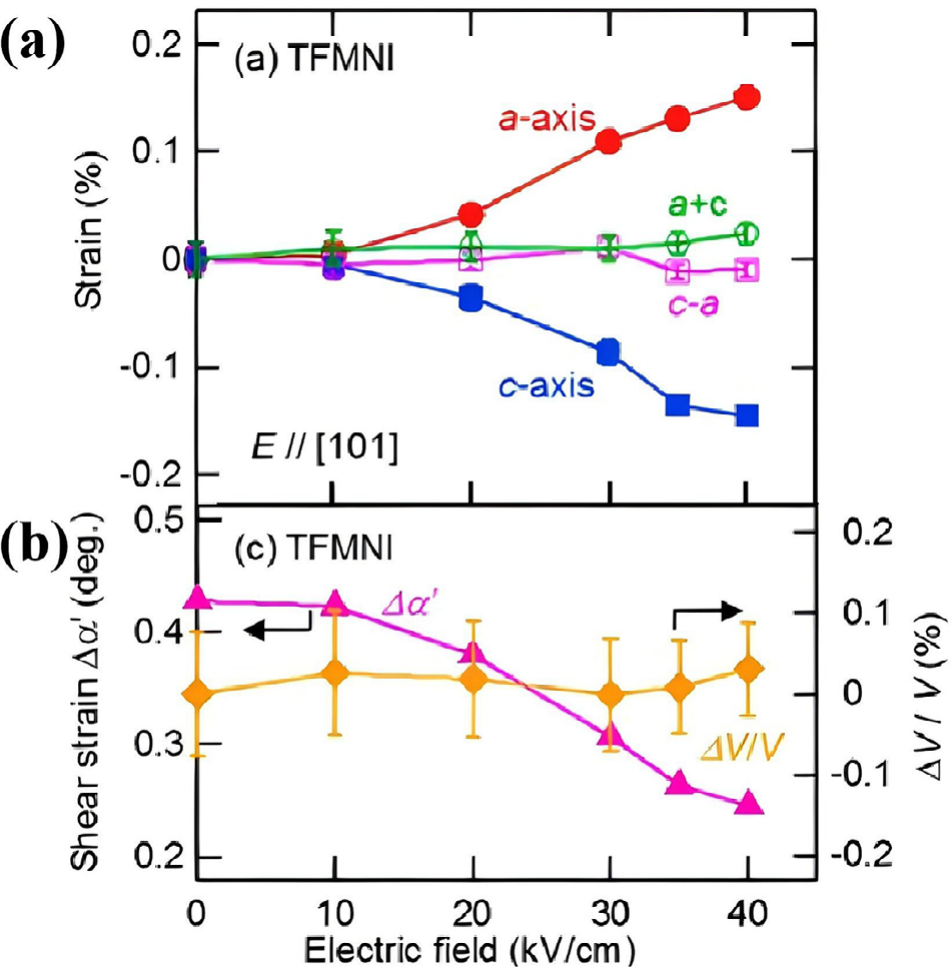
(a) Lattice strain along different directions in a TFMNI under different electric fields; The error bars are smaller than the symbols; (b) Electric field dependence of the shear strain ∆*α*’ and cell volume variation ∆*V*. Reproduced with permission [31]. Copyright 2018, American Chemical Society.

The field‐induced phase transition from the antiferroelectric to the ferroelectric phase under an applied electric field involves the rearrangement of the crystal lattice and transformation of the structural symmetry, accompanied by obvious lattice strain, that is, the electrostrictive effect. Kensuke Kobayashi et al. investigated the lattice strain of TFMNI crystals under an applied electric field. At an external electric field of 25 kV/cm, the lattice strain of TFMNI crystals was about 0.07 % along the *a*‐axis [31]. When the applied electric field is further increased to 40 kV/cm, the TFMNI crystal manifests a lattice strain of up to 0.15 % (Figure 16a). The strain corresponds to an equivalent piezoelectric coefficient of up to 376 pm/V, which is an order of magnitude larger than the longitudinal piezoelectric coefficient *d*_33_ (31 pm/V) of polyvinylidene fluoride (PVDF) and comparable to the *d*_33_ coefficient about 370 pm/V of commonly used piezoelectric ceramics. Meanwhile, as shown in Figure 16b, with the increase of the field magnitude, ∆α’ decreased toward zero, whereas the variation of the unit cell volume ∆*V*/*V* remained negligibly small. Combined with the analysis of the crystal structure under the electric field, the huge electrostrictive effect results from the shear strain produced during the polarity reversal of the hydrogen bond chain. The study demonstrated the structural response mechanism of organic antiferroelectrics driven by electric fields, showing potential for applications of electromechanical conversion.

### 3.4 Energy Storage Properties

The typical AFE–FE phase transitions under an external electric field, coupled with the significant changes in polarization, enable a large amount of energy to be stored and released, which endows AFEs with great potential for energy storage [9, 54, 55, 56, 57]. Molecular‐based antiferroelectric materials exhibit unique polarization response behavior, where the dynamic movement or orientation of organic cations can be controlled by an external electric field, thereby enabling the occurrence of antiferroelectric‐ferroelectric phase transitions and the storage and release of energy. Excitingly, the coercive field of molecular antiferroelectrics is generally low, and the antiferroelectric‐ferroelectric reversible phase transition can be triggered at a lower electric field, providing a new material path for the next generation of highly efficient dielectric energy storage devices. Meanwhile, the high structural tunability of molecular antiferroelectrics enables further optimization of the polarization magnitude and phase transition behavior through rational molecular design, thereby optimizing energy storage performance.

**FIGURE 17:**
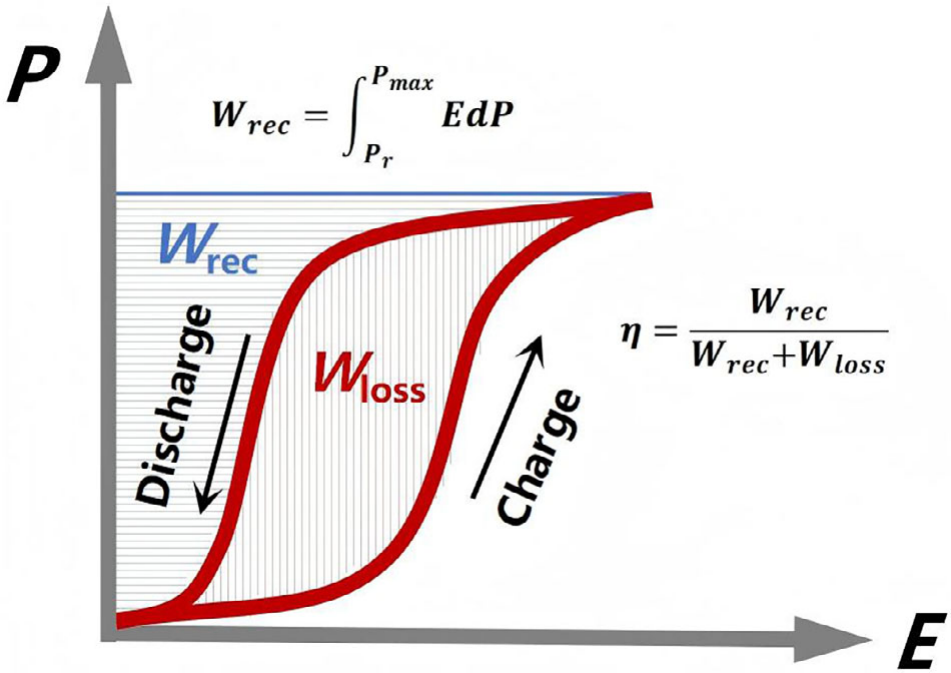
Schematic diagram for the calculation of energy storage properties of antiferroelectrics.

**FIGURE 18:**
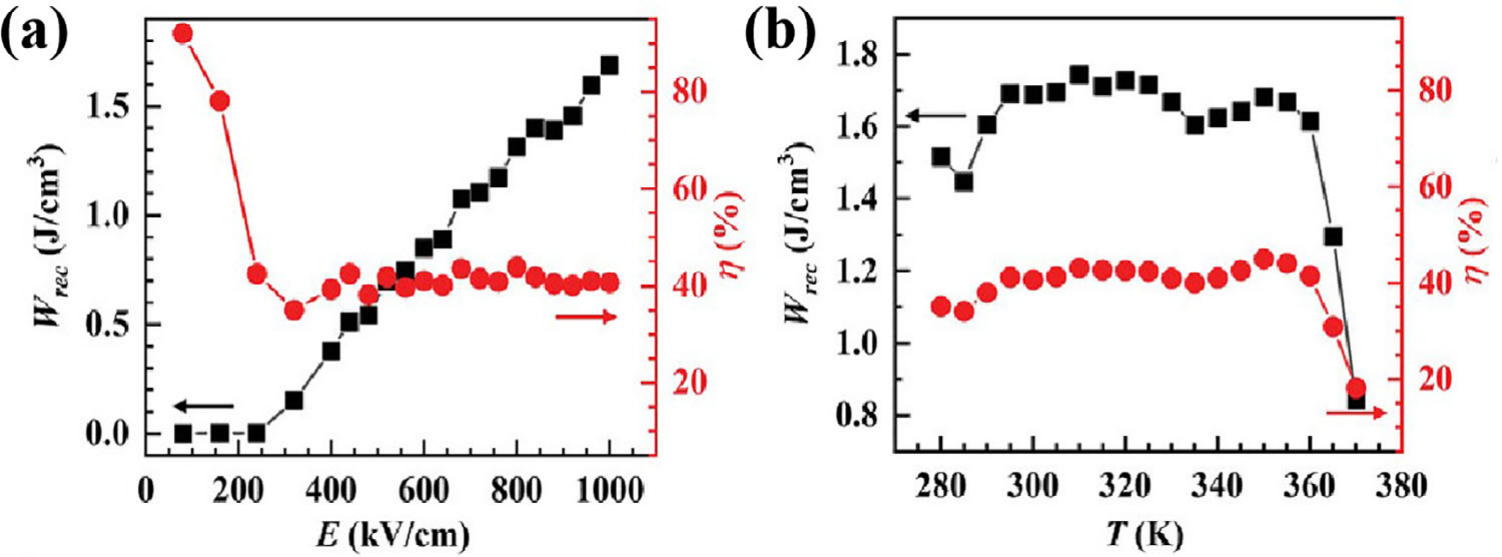
The recoverable energy density (*W*_rec_) and energy efficiency (*η*) as a function of electric field (b) and temperature (c) of (PMA)_2_PbBr_4_. Reproduced with permission [47]. Copyright 2023, Wiley‐VCH.

As depicted in Figure 17, the recoverable energy density (*W*_rec_) and energy efficiency (*η*), which are important metrics to appraise the energy storage performances for practical applications, can be estimated from the hysteresis loops. The energy storage efficiency of (i‐BA)_2_CsPb_2_Br_7_ is calculated to be 69 %, while the computational value of *W*_rec_ is 0.26 J/cm^3^ at room temperature [39]. Significantly, the *η* values of (i‐BA)_2_CsPb_2_Br_7_ show good thermal stability during the whole antiferroelectric temperature range. The high structural tunability of hybrid perovskite molecular antiferroelectrics allows for further optimization of the polarization strength and phase transition characteristics through molecular design, thereby optimizing dielectric energy storage performance. For example, (i‐BA)_2_(FA)Pb_2_Br_7_ affords an ultrahigh storage efficiency of ∼91 % at a considerably low critical electric field (*E*_cr_ = 41 kV/cm), which can be attributed to the much lower field hysteresis of 47 kV/cm [40]. Meanwhile, the energy storage performance of (i‐BA)_2_(FA)Pb_2_Br_7_ demonstrates significant frequency stability, where the *P*_max_ value slightly decreases, and ∆*E* increases with the frequency ranging from 100 to 40 Hz. The high‐quality single crystals and films of 2D hybrid perovskite antiferroelectric (PMA)_2_PbBr_4_ were prepared, of which the ferroelectric, dielectric, and energy storage properties were systematically studied [47]. Excitingly, (PMA)_2_PbBr_4_ exhibited breakdown electric fields over 1000 kV/cm, energy storage densities up to 1.7 J/cm^3^, and operating temperatures ranging up to 70 K (Figure 18). The study demonstrates that (PMA)_2_PbBr_4_, while maintaining the advantages of the low‐temperature solution fabrication method, shows both high energy storage density and high breakdown field strength, highlighting the significant potential of hybrid perovskite molecular‐antiferroelectrics for high‐performance energy storage. Table 2 systematically compares the key energy storage performance parameters of the reported hybrid perovskite molecular antiferroelectric. These molecular‐based materials demonstrate energy storage capabilities that rival those of their inorganic counterparts. Remarkably, a key advantage is their ability to deliver high energy efficiency at low operating electric fields, underscoring their potential for low‐power and highly efficient energy storage applications.

**TABLE 2:** Comparison of the crucial energy storage parameters of the reported molecular antiferroelectrics and some inorganic antiferroelectrics.

Compound	E (kV cm^−1^)	*η* (%)	*W*_rec_ (J cm^−3^)	Refs.
TFMBI	22	44	0.061	[26]
DFMBI	86	78	0.528	[26]
TCMBI	81	62	0.333	[26]
SQA	151	94	1.44	[32]
CMC	66	52	0.28	[51]
F‐PEAB	70	83.6	0.096	[33]
[H‐55dmbp][Hca]	173	90	0.51	[32]
(i‐BA)_2_CsPb_2_Br_7_	94	69	0.26	[39]
(i‐BA)_2_(FA)Pb_2_Br_7_	41	91	0.13	[40]
(PMA)_2_PbBr_4_	1000	41	1.7	[47]
(i‐BA)_2_CsGe_2_I_7_	22.5	79.76	0.27	[41]
(CHMA)_2_CsAgBiBr_7_	145–205	∼63	∼0.33	[46]
(Pb_0.965_La_0.035_)(Zr_0.9_Ti_0.1_)_0.99125_O_3_	65	65	1.85	[58]
Pb_0.97_La_0.02_(Zr_0.58_Sn_0.35_T_0.07_)O_3_	115	86.1	2.35	[59]
AgNbO_3_	175	40	2.1	[60]
AgNbO_3_‐0.1 wt. %WO_3_	200	50	3.3	[61]

### 3.5 Electrocaloric Effect

Electrocaloric cooling, inducing dipole entropy change by an electric field in ferroelectrics, has emerged as a forward‐looking solid‐state cooling technology [62, 63, 64]. The internal electric dipole flips, and the orientation freedom decreases as the electric field is loaded/unloaded, causing an increase or decrease in dipole entropy and lattice entropy, resulting in a temperature change in the ferroelectrics. Heat transfer is achieved through heat absorption and release behavior during the process. Based on the Maxwell relationship, the reversible electrocaloric change Δ*T* and isothermal entropy change Δ*S* due to a change in external electric field under adiabatic conditions can be determined by:

$$\Delta S=-\int_{E_{1}}^{E_{2}}\left(\frac{\Delta P}{\Delta T}\right)dE$$

$$\Delta T=-\int_{E_{1}}^{E_{2}}\frac{T}{\rho c_{p}}\left(\frac{\Delta P}{\Delta T}\right)dE$$

where *T* is the temperature, *P* is the polarization, ρ is the mass density, which depends on both the electric field and temperature.

Thus, electrocaloric materials often exhibit the largest response near their phase transitions, where the thermally driven changes in measured polarization are largest [65]. Within this context, the rich phase‐transition behavior and multiple polarization changes of molecular‐based antiferroelectric materials provide conditions for electrocaloric cooling and ultrahigh EC strength.

**FIGURE 19:**
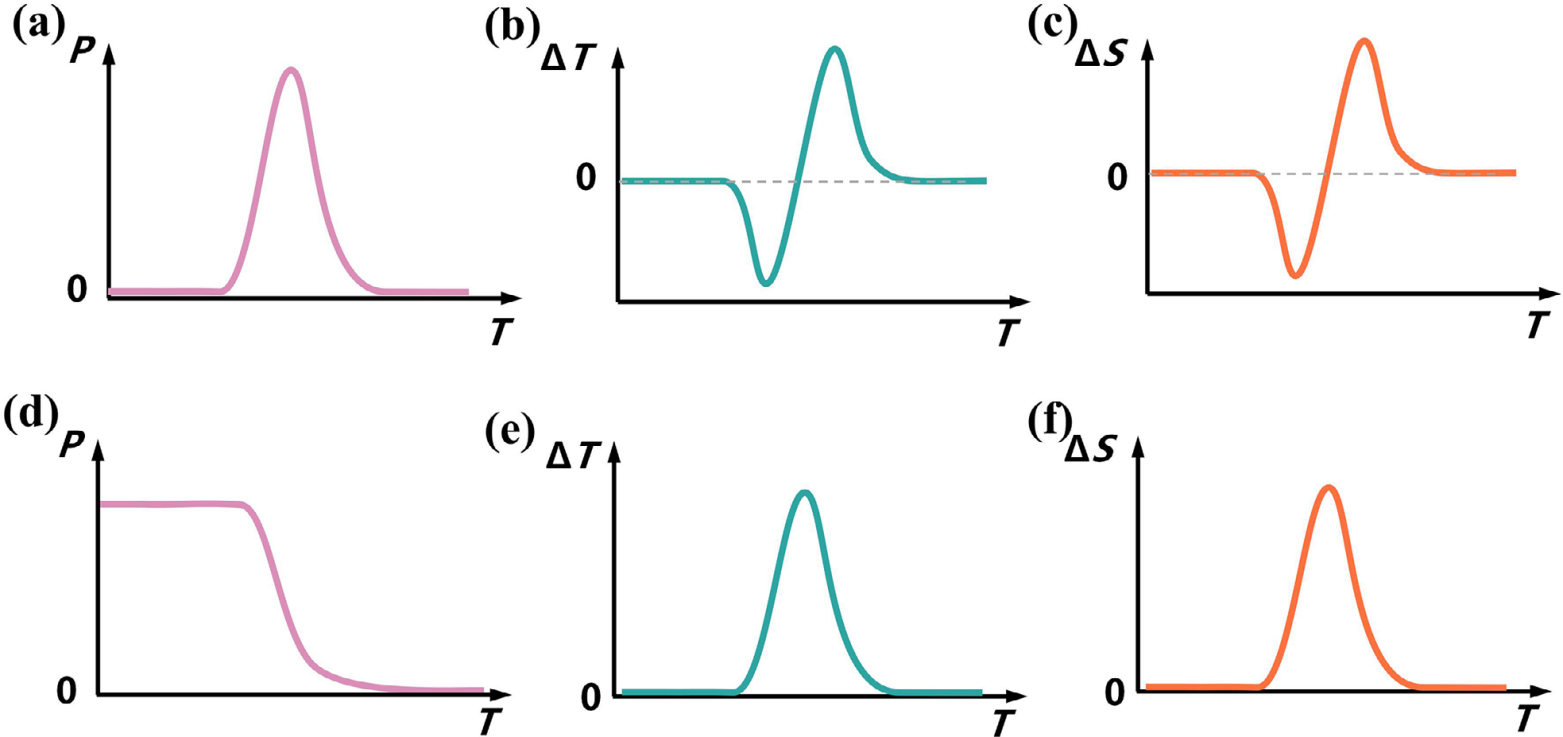
Diagram of temperature‐dependent polarization in molecular AFEs with FE‐AFE‐PE transitions and the corresponding reversible electrocaloric change Δ*T* and isothermal entropy change Δ*S*; Diagram of temperature‐dependent polarization in molecular AFEs with AFE‐FE‐PE transitions and the corresponding Δ*T* and Δ*S*.

**FIGURE 20:**
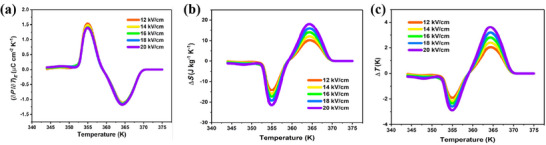
Temperature dependence of pyroelectric coefficient(a), isothermal entropy change (Δ*S*), and (b) EC temperature change (Δ*T*) (c) under different electric fields in CMB. Reproduced with permission [50]. Copyright 2021American Chemical Society.

In particular, the structural consecutive phase transition of antiferroelectric‐ferroelectric‐paraelectric molecular antiferroelectrics as the temperature rises is accompanied by polarization from absent to present and then back to absent, enabling both negative and positive electrocaloric effects (Figure 19a–c). For instance, the reorientation of organic cations and relative displacement of anionic moieties drive the successive AFE‐FE‐PE phase transitions at 364 and 368 K in CMB [50]. From the experimental *P*‐*E* hysteresis loops at different temperatures, the ∂*P*/∂*T* curve was obtained, and the electrocaloric‐related parameters were calculated. Under a relatively small electric field ∼20 kV/cm, the positive and negative maximum Δ*S* values are calculated as 20.8 and −22.7 J/(kg K), respectively, while the temperature changes (Δ*T*) of 4.2 and ‐3 K are achieved (Figure 20). Such a presence of both positive and negative EC responses under a relatively low electric field is quite scarce in current reports, which encourages the further exploration of new molecular AFEs for environmentally friendly solid‐state refrigeration device applications.

Meanwhile, the structural consecutive phase transition of ferroelectric‐antiferroelectric‐paraelectric as the temperature rises is accompanied by polarization from present to absent near the phase transition, enabling positive electrocaloric effects (Figure 19d–f). For example, upon application of an electric field (Δ*E*) of 13 kV/cm, the maximal values of ΔS and Δ*T* of (i‐AA)_2_CsPb_2_Br_7_ are calculated to be 4.1 J K^−1^ kg^−1^ and 2.0 K, respectively [45]. Note that the largest EC response occurs in the near‐room‐temperature range, which is highly desirable in high‐efficiency solid‐state refrigeration. Most fascinatingly, single crystals of (i‐AA)_2_CsPb_2_Br_7_ demonstrate giant near room‐temperature EC strength merits with a high Δ*S*/Δ*E* of 3.15 J m kg^−1^ K^−1^ MV^−1^ and Δ*T*/Δ*E* of 15.4 K m MV^−1^. Distinct from those conventional ferroelectric polymers requiring a quite high external electric field [66, 67], the polarization switching in (i‐AA)_2_CsPb_2_Br_7_ can be easily fulfilled under a considerably small electric field (only ∼5 kV/cm), which is sufficient to induce a remarkable EC response. Table 3 shows a comparison of the electrocaloric effect of the reported molecular antiferroelectrics and some inorganic antiferroelectrics. These molecular‐based antiferroelectrics exhibit electrocaloric cooling performance that is comparable to that of inorganic antiferroelectric ceramics, which can be obtained at low applied electric fields. This low driving field is crucial as it contributes to enhancing the EC strength, which is also a key and significant parameter for practical cooling devices.

**TABLE 3:** Comparison of the electrocaloric effect of the reported molecular antiferroelectrics and some inorganic antiferroelectrics.

Compound	Δ*E* (kV cm^−1^)	Δ*S* (J kg^−1^ K^−1^)	Δ*T* (K)	Ref.
(IA)_2_CsPb_2_Br_7_	13	4.1	2	[45]
(3‐pyrrolinium)CdBr_3_	7.41	1.18	—	[48]
CMB	20	−22.7, 20.8	−3, 4.2	[42]
(i‐BA)_2_(EA)_2_Pb_3_I_10_	18	−33.3	−11.7	[44]
BaTiO_3_ crystal	12	—	0.9	[52]
PbZrO_3_ thin films	500	−8	−6.8	[68]
(Pb_0.97_La_0.02_)(Zr_0.8_Sn_0.14_Ti_0.06_)O_3_	110	−12.5	−11.5	[69]
La‐doped PbZrO_3_	308		−5	[70]
Eu‐Doped PbZrO_3_	709	−5.42	−6.62	[71]
PbMg_0.5_W_0.5_O_3_	120	1.68, −1.93	1.79, −2.02	[14]
Hf_0.5_Zr_0.5_O_2_	3260	−10.9	−10.8	[66]
Bi_0.5_Na_0.5_TiO_3_	70		−1.62	[72]

## 4 Conclusion and Prospects

This review provides a systematic overview of recent advances in the design and application of molecular antiferroelectrics, establishing a strategic framework to guide future research and development of such an emerging material system. As a rising star, molecular antiferroelectrics have come into our sight due to their excellent antiferroelectric properties and natural advantages of structural softness and lightweight, meeting the requirements of integration and miniaturization for the next generation of electronic devices.

Extensive research work shows that the development of molecular antiferroelectrics is still in its infancy compared to traditional antiferroelectrics. Although several molecular antiferroelectrics showing considerable performance have been developed, it is still a long way from commercialization. We believe that further research on molecular antiferroelectrics can be conducted in the following directions to promote its early application.

### 4.1 Optimization of New Molecular Antiferroelectrics

Up to now, the antiferroelectric properties of most of the reported molecular ferroelectrics are still much inferior to those of perovskite oxide antiferroelectrics, which promotes the innovative strategies to improve the performance of molecular antiferroelectrics.

The strategic enhancement of lone‐pair expression represents a frontier in the molecular‐level design of high‐performance AFEs. There has been an upsurge in the research on Germanium‐based perovskites, in which the lone‐pair stereochemical activity in Ge^2+^ makes a significant contribution to the large structural distortion and giant polarization [41, 73, 74, 75]. Fine‐tuning the local environment of the lone‐pair cation and amplifying lone‐pair effects would induce a greater off‐center displacement of ions, thereby leading to a significant increase in field‐induced polarization.

Building on the well‐established developments of solid solutions in inorganic oxide antiferroelectrics, this strategy holds immense promise for molecular systems [76, 77, 78]. The structural flexibility endows molecular antiferroelectrics with a large scope for compositional engineering to accommodate different molecular building blocks, which allows for the fine‐tuning of key antiferroelectric properties such as field polarization, switching field, and Curie temperature. Additionally, the investigation of compositional phase diagrams and the identification of a morphotropic phase boundary are crucial in the development of molecular antiferroelectrics

Moreover, most hybrid halide perovskite molecular antiferroelectrics suffer from the toxicity of lead, raising environmental concerns. More efforts to develop environmentally friendly antiferroelectrics are highly expected. Meanwhile, molecular antiferroelectrics suffer from insufficient environmental stability primarily, manifesting as structural degradation or performance decay under humidity, high temperature, or oxygen exposure. Developing strategies, including hydrophobic engineering and lattice reinforcement, is highly impressive.

### 4.2 Integration of New Functionality of Molecular Antiferroelectrics

The ability of piezoelectrics and ferroelectrics to generate above‐bandgap photovoltages involving the intrinsic asymmetry of the lattice has stimulated scientists for many decades. A suitably large external field can cause a transition from their antipolar ground state to a polar phase in antiferroelectrics, in which a switching under illumination results in the pinning of the polar state and generates above‐bandgap photovoltages [79]. Halide perovskite antiferroelectrics with excellent semiconducting properties are expected to emerge in the photoelectronic field, manifesting fascinating photoelectronic functionalities.

The recent discovery of optical control over polarization switching in ferroelectric materials opens a compelling avenue for exploring analogous phenomena in AFEs [80]. Building on the mechanisms observed in ferroelectrics, such as photostrictive effects and photo‐carrier modulation of depolarization fields, light illumination could potentially be used to selectively destabilize the antipolar order in AFEs. The photomodulation of polarization states enables the targeted enhancement of functional properties in antiferroelectric materials. Meanwhile, the optical modulation of polarization would enable low‐power and non‐contact control over their electronic devices.

### 4.3 Exploration of New Applications of Molecular Antiferroelectrics

Antiferroelectric molecular materials, particularly those with “soft” characteristics, offer unprecedented opportunities for next‐generation flexible devices due to their molecular‐level compliance, mechanical robustness, and rich phase‐transition behavior.

The unique electric‐field‐induced antiferroelectric‐to‐ferroelectric phase transition, accompanied by significant strain, endows molecular antiferroelectrics with an exceptional platform for flexible large‐strain micro‐actuators and mechanical energy harvesters. Their inherently compliant lattice dramatically lowers the energy barrier for this phase transformation, enabling substantial, reversible volume changes at relatively low operating voltages, which is highly desirable in energy‐efficient systems.

Encouraged by advances in ferroelectric synapses for neuromorphic computing [81], antiferroelectrics present a compelling frontier. Their field‐induced phase transition enables analog conductance modulation through progressive domain switching, which provides inherent advantages for achieving symmetric and linear weight updates. The field‐induced phase transition offers a novel physical mechanism for synaptic weight modulation, while the absence of remnant polarization in the ground state ensures low static power consumption. This unique combination positions AFEs as a promising platform for developing high‐fidelity and energy‐efficient artificial neural networks.

### 4.4 Large‐Area Fabrication of Molecular Antiferroelectrics for Electronic Devices

The large‐area fabrication of high‐quality molecular antiferroelectric materials represents a pivotal milestone in bridging the gap between fundamental discovery and practical deployment in electronic devices. The inherent fragility and anisotropic nature of low‐dimensional molecular antiferroelectric crystals often limit their growth to millimeter‐sized specimens. Techniques, such as spatially confined crystallization and seeded epitaxial growth, for growing crystals with large dimensions should be developed.

Meanwhile, advanced thin‐film fabrication techniques need to be developed to precisely control the directional assembly of molecules over areas of tens to hundreds of square centimeters.

## Funding

This work was supported by the National Natural Science Foundation of China (52473283, 22193042, 22125110, and 22435005), the Strategic Priority Research Program of the Chinese Academy of Sciences (XDB1170000), the Self‐deployed Key Project of State Key Laboratory of Functional Crystals and Devices (GNJT‐2025‐ZD01), the Natural Science Foundation of Fujian Province (2024J010037) and the Self‐deployment Project Research Program of Haixi Institutes, Chinese Academy of Sciences (CXZX‐2023‐JQ04).

## Conflicts of Interest

The authors have nothing to report.

## Data Availability

The data that support the findings of this study are available from the corresponding author upon reasonable request.
